# Supplementary material for: Pyruvate Dehydrogenase Kinase 1 inhibition mediated oxidative phosphorylation enhancement in cartilage promotes osteoarthritis progression
Source: BMC Musculoskelet Disord. 2023 Jul 20;24:597. doi: 10.1186/s12891-023-06585-6 (PMC10357736; doi:10.1186/s12891-023-06585-6)
Supplement: Supplementary file 1 — Additional file 1. [file 12891_2023_6585_MOESM1_ESM.zip › KEGG.Gsea.1653622432464/KEGG_PANCREATIC_CANCER.html]

Details for gene set KEGG\_PANCREATIC\_CANCER[GSEA]

|  || Dataset | 55457\_collapsed\_to\_symbols.cls.cls #OA\_versus\_normal.cls.cls #OA\_versus\_normal\_repos |
| Phenotype | cls.cls#OA\_versus\_normal\_repos |
| Upregulated in class | normal |
| GeneSet | KEGG\_PANCREATIC\_CANCER |
| Enrichment Score (ES) | -0.251456 |
| Normalized Enrichment Score (NES) | -1.015801 |
| Nominal p-value | 0.43383583 |
| FDR q-value | 0.62273484 |
| FWER p-Value | 1.0 |
Table: GSEA Results Summary

  

Fig 1: Enrichment plot: KEGG\_PANCREATIC\_CANCER      
 Profile of the Running ES Score & Positions of GeneSet Members on the Rank Ordered List

  

| SYMBOL | TITLE | RANK IN GENE LIST | RANK METRIC SCORE | RUNNING ES | CORE ENRICHMENT || 1 | RALBP1 | ralA binding protein 1 [Source:HGNC Symbol;Acc:HGNC:9841] | 217 | 0.808 | 0.0194 | No |
| 2 | SMAD2 | SMAD family member 2 [Source:HGNC Symbol;Acc:HGNC:6768] | 275 | 0.768 | 0.0495 | No |
| 3 | MAPK9 | mitogen-activated protein kinase 9 [Source:HGNC Symbol;Acc:HGNC:6886] | 721 | 0.588 | 0.0413 | No |
| 4 | MAPK3 | mitogen-activated protein kinase 3 [Source:HGNC Symbol;Acc:HGNC:6877] | 757 | 0.579 | 0.0646 | No |
| 5 | CDKN2A | cyclin dependent kinase inhibitor 2A [Source:HGNC Symbol;Acc:HGNC:1787] | 799 | 0.570 | 0.0870 | No |
| 6 | ERBB2 | erb-b2 receptor tyrosine kinase 2 [Source:HGNC Symbol;Acc:HGNC:3430] | 963 | 0.526 | 0.0980 | No |
| 7 | AKT1 | AKT serine/threonine kinase 1 [Source:HGNC Symbol;Acc:HGNC:391] | 1192 | 0.478 | 0.1017 | No |
| 8 | CDK4 | cyclin dependent kinase 4 [Source:HGNC Symbol;Acc:HGNC:1773] | 1375 | 0.447 | 0.1077 | No |
| 9 | RAD51 | RAD51 recombinase [Source:HGNC Symbol;Acc:HGNC:9817] | 1581 | 0.416 | 0.1104 | No |
| 10 | PIK3CB | "phosphatidylinositol-4,5-bisphosphate 3-kinase catalytic subunit beta [Source:HGNC Symbol;Acc:HGNC:8976]" | 2186 | 0.340 | 0.0786 | No |
| 11 | VEGFC | vascular endothelial growth factor C [Source:HGNC Symbol;Acc:HGNC:12682] | 2239 | 0.335 | 0.0896 | No |
| 12 | EGF | epidermal growth factor [Source:HGNC Symbol;Acc:HGNC:3229] | 2399 | 0.315 | 0.0914 | No |
| 13 | TGFB1 | transforming growth factor beta 1 [Source:HGNC Symbol;Acc:HGNC:11766] | 2490 | 0.303 | 0.0980 | No |
| 14 | RALB | RAS like proto-oncogene B [Source:HGNC Symbol;Acc:HGNC:9840] | 3224 | 0.228 | 0.0511 | No |
| 15 | BAD | BCL2 associated agonist of cell death [Source:HGNC Symbol;Acc:HGNC:936] | 3788 | 0.178 | 0.0153 | No |
| 16 | RAC3 | Rac family small GTPase 3 [Source:HGNC Symbol;Acc:HGNC:9803] | 3849 | 0.171 | 0.0183 | No |
| 17 | BRCA2 | BRCA2 DNA repair associated [Source:HGNC Symbol;Acc:HGNC:1101] | 3853 | 0.170 | 0.0257 | No |
| 18 | RELA | "RELA proto-oncogene, NF-kB subunit [Source:HGNC Symbol;Acc:HGNC:9955]" | 3942 | 0.161 | 0.0261 | No |
| 19 | SMAD3 | SMAD family member 3 [Source:HGNC Symbol;Acc:HGNC:6769] | 4061 | 0.150 | 0.0236 | No |
| 20 | STAT1 | signal transducer and activator of transcription 1 [Source:HGNC Symbol;Acc:HGNC:11362] | 4495 | 0.113 | -0.0051 | No |
| 21 | NFKB1 | nuclear factor kappa B subunit 1 [Source:HGNC Symbol;Acc:HGNC:7794] | 4626 | 0.102 | -0.0106 | No |
| 22 | E2F1 | E2F transcription factor 1 [Source:HGNC Symbol;Acc:HGNC:3113] | 5137 | 0.061 | -0.0476 | No |
| 23 | TGFBR1 | transforming growth factor beta receptor 1 [Source:HGNC Symbol;Acc:HGNC:11772] | 5280 | 0.050 | -0.0565 | No |
| 24 | TP53 | tumor protein p53 [Source:HGNC Symbol;Acc:HGNC:11998] | 5511 | 0.032 | -0.0730 | No |
| 25 | IKBKB | inhibitor of nuclear factor kappa B kinase subunit beta [Source:HGNC Symbol;Acc:HGNC:5960] | 5914 | 0.001 | -0.1042 | No |
| 26 | RB1 | RB transcriptional corepressor 1 [Source:HGNC Symbol;Acc:HGNC:9884] | 6013 | -0.006 | -0.1116 | No |
| 27 | RAC2 | Rac family small GTPase 2 [Source:HGNC Symbol;Acc:HGNC:9802] | 6098 | -0.013 | -0.1176 | No |
| 28 | RAF1 | "Raf-1 proto-oncogene, serine/threonine kinase [Source:HGNC Symbol;Acc:HGNC:9829]" | 6187 | -0.019 | -0.1236 | No |
| 29 | TGFB3 | transforming growth factor beta 3 [Source:HGNC Symbol;Acc:HGNC:11769] | 6293 | -0.027 | -0.1306 | No |
| 30 | BCL2L1 | BCL2 like 1 [Source:HGNC Symbol;Acc:HGNC:992] | 6698 | -0.055 | -0.1596 | No |
| 31 | MAPK1 | mitogen-activated protein kinase 1 [Source:HGNC Symbol;Acc:HGNC:6871] | 6853 | -0.068 | -0.1685 | No |
| 32 | TGFB2 | transforming growth factor beta 2 [Source:HGNC Symbol;Acc:HGNC:11768] | 6985 | -0.079 | -0.1752 | No |
| 33 | JAK1 | Janus kinase 1 [Source:HGNC Symbol;Acc:HGNC:6190] | 6995 | -0.079 | -0.1723 | No |
| 34 | RAC1 | Rac family small GTPase 1 [Source:HGNC Symbol;Acc:HGNC:9801] | 7192 | -0.096 | -0.1833 | No |
| 35 | PIK3CD | "phosphatidylinositol-4,5-bisphosphate 3-kinase catalytic subunit delta [Source:HGNC Symbol;Acc:HGNC:8977]" | 7405 | -0.111 | -0.1949 | No |
| 36 | CCND1 | cyclin D1 [Source:HGNC Symbol;Acc:HGNC:1582] | 7512 | -0.119 | -0.1977 | No |
| 37 | PIK3CA | "phosphatidylinositol-4,5-bisphosphate 3-kinase catalytic subunit alpha [Source:HGNC Symbol;Acc:HGNC:8975]" | 7733 | -0.136 | -0.2088 | No |
| 38 | ARHGEF6 | Rac/Cdc42 guanine nucleotide exchange factor 6 [Source:HGNC Symbol;Acc:HGNC:685] | 7900 | -0.147 | -0.2151 | No |
| 39 | PIK3CG | "phosphatidylinositol-4,5-bisphosphate 3-kinase catalytic subunit gamma [Source:HGNC Symbol;Acc:HGNC:8978]" | 7953 | -0.152 | -0.2123 | No |
| 40 | AKT3 | AKT serine/threonine kinase 3 [Source:HGNC Symbol;Acc:HGNC:393] | 8008 | -0.156 | -0.2095 | No |
| 41 | E2F2 | E2F transcription factor 2 [Source:HGNC Symbol;Acc:HGNC:3114] | 8090 | -0.162 | -0.2085 | No |
| 42 | ARAF | "A-Raf proto-oncogene, serine/threonine kinase [Source:HGNC Symbol;Acc:HGNC:646]" | 8092 | -0.163 | -0.2013 | No |
| 43 | CASP9 | caspase 9 [Source:HGNC Symbol;Acc:HGNC:1511] | 8142 | -0.166 | -0.1976 | No |
| 44 | BRAF | "B-Raf proto-oncogene, serine/threonine kinase [Source:HGNC Symbol;Acc:HGNC:1097]" | 8171 | -0.169 | -0.1922 | No |
| 45 | TGFBR2 | transforming growth factor beta receptor 2 [Source:HGNC Symbol;Acc:HGNC:11773] | 8241 | -0.174 | -0.1898 | No |
| 46 | CDK6 | cyclin dependent kinase 6 [Source:HGNC Symbol;Acc:HGNC:1777] | 8393 | -0.185 | -0.1932 | No |
| 47 | STAT3 | signal transducer and activator of transcription 3 [Source:HGNC Symbol;Acc:HGNC:11364] | 8453 | -0.190 | -0.1893 | No |
| 48 | VEGFB | vascular endothelial growth factor B [Source:HGNC Symbol;Acc:HGNC:12681] | 8673 | -0.206 | -0.1971 | No |
| 49 | TGFA | transforming growth factor alpha [Source:HGNC Symbol;Acc:HGNC:11765] | 8748 | -0.212 | -0.1933 | No |
| 50 | PIK3R3 | phosphoinositide-3-kinase regulatory subunit 3 [Source:HGNC Symbol;Acc:HGNC:8981] | 8801 | -0.217 | -0.1876 | No |
| 51 | PIK3R5 | phosphoinositide-3-kinase regulatory subunit 5 [Source:HGNC Symbol;Acc:HGNC:30035] | 8804 | -0.217 | -0.1780 | No |
| 52 | MAPK8 | mitogen-activated protein kinase 8 [Source:HGNC Symbol;Acc:HGNC:6881] | 8956 | -0.228 | -0.1795 | No |
| 53 | SMAD4 | SMAD family member 4 [Source:HGNC Symbol;Acc:HGNC:6770] | 9035 | -0.235 | -0.1750 | No |
| 54 | PLD1 | phospholipase D1 [Source:HGNC Symbol;Acc:HGNC:9067] | 9365 | -0.261 | -0.1889 | No |
| 55 | MAPK10 | mitogen-activated protein kinase 10 [Source:HGNC Symbol;Acc:HGNC:6872] | 9699 | -0.291 | -0.2018 | No |
| 56 | MAP2K1 | mitogen-activated protein kinase kinase 1 [Source:HGNC Symbol;Acc:HGNC:6840] | 10197 | -0.340 | -0.2252 | No |
| 57 | RALGDS | ral guanine nucleotide dissociation stimulator [Source:HGNC Symbol;Acc:HGNC:9842] | 10287 | -0.349 | -0.2164 | No |
| 58 | KRAS | "KRAS proto-oncogene, GTPase [Source:HGNC Symbol;Acc:HGNC:6407]" | 10544 | -0.377 | -0.2194 | No |
| 59 | PIK3R2 | phosphoinositide-3-kinase regulatory subunit 2 [Source:HGNC Symbol;Acc:HGNC:8980] | 10956 | -0.425 | -0.2323 | Yes |
| 60 | IKBKG | inhibitor of nuclear factor kappa B kinase regulatory subunit gamma [Source:HGNC Symbol;Acc:HGNC:5961] | 10986 | -0.428 | -0.2153 | Yes |
| 61 | VEGFD | vascular endothelial growth factor D [Source:HGNC Symbol;Acc:HGNC:3708] | 11095 | -0.443 | -0.2038 | Yes |
| 62 | PIK3R1 | phosphoinositide-3-kinase regulatory subunit 1 [Source:HGNC Symbol;Acc:HGNC:8979] | 11099 | -0.443 | -0.1841 | Yes |
| 63 | CHUK | component of inhibitor of nuclear factor kappa B kinase complex [Source:HGNC Symbol;Acc:HGNC:1974] | 11624 | -0.521 | -0.2016 | Yes |
| 64 | EGFR | epidermal growth factor receptor [Source:HGNC Symbol;Acc:HGNC:3236] | 12109 | -0.622 | -0.2113 | Yes |
| 65 | RALA | RAS like proto-oncogene A [Source:HGNC Symbol;Acc:HGNC:9839] | 12398 | -0.707 | -0.2019 | Yes |
| 66 | VEGFA | vascular endothelial growth factor A [Source:HGNC Symbol;Acc:HGNC:12680] | 12629 | -0.813 | -0.1833 | Yes |
| 67 | CDC42 | cell division cycle 42 [Source:HGNC Symbol;Acc:HGNC:1736] | 12681 | -0.852 | -0.1490 | Yes |
| 68 | PGF | placental growth factor [Source:HGNC Symbol;Acc:HGNC:8893] | 12850 | -1.059 | -0.1144 | Yes |
| 69 | E2F3 | E2F transcription factor 3 [Source:HGNC Symbol;Acc:HGNC:3115] | 12886 | -1.224 | -0.0621 | Yes |
| 70 | AKT2 | AKT serine/threonine kinase 2 [Source:HGNC Symbol;Acc:HGNC:392] | 12896 | -1.404 | 0.0003 | Yes |
Table: GSEA details [plain text format]

  

Fig 2: KEGG\_PANCREATIC\_CANCER      
 Blue-Pink O' Gram in the Space of the Analyzed GeneSet

  

Fig 3: KEGG\_PANCREATIC\_CANCER: Random ES distribution      
 Gene set null distribution of ES for **KEGG\_PANCREATIC\_CANCER**
